# Application of Multiplanar Volume Reconstruction Technique for the Assessment of Electrode Location and Analysis of the Correlation to Cochlear Programming and Performance in Common Cavity Deformity

**DOI:** 10.3389/fneur.2021.783225

**Published:** 2022-01-11

**Authors:** Xingmei Wei, Huaiyu Zhang, Simeng Lu, Mengge Yang, Biao Chen, Jingyuan Chen, Lifang Zhang, Sha Liu, Junfang Xian, Yongxin Li, Ying Kong

**Affiliations:** ^1^Department of Otorhinolaryngology Head and Neck Surgery, Beijing Tongren Hospital, Capital Medical University, Beijing, China; ^2^Key Laboratory of Otolaryngology Head and Neck Surgery, Capital Medical University, Ministry of Education, Beijing, China; ^3^Department of Radiology, Beijing Tongren Hospital, Capital Medical University, Beijing, China; ^4^Beijing Institute of Otolaryngology, Capital Medical University, Ministry of Education, Beijing, China

**Keywords:** cochlear implantation, common cavity deformity, multiplanar volume reconstructions, maximum comfortable level, auditory outcomes

## Abstract

**Purpose:** Owing to the characteristic anatomy, cochlear implantation (CI) for common cavity deformity (CCD) has resulted in varied outcomes and frequent facial and vestibular nerve stimulation. The current study analyzed the correlation among the distance between each electrode and cavity wall (abbreviation, D), programming parameters, and performances outcomes.

**Materials and Methods:** The current, retrospective study included 25 patients (27 ears) with CCD underwent CI. The multiplanar volume reconstruction (MPVR) techniques were employed to reconstruct and evaluate the postoperative temporal bone CT. The D and maximum comfortable level (MCL) 6 months after CI, facial and vestibular nerve stimulation, and outcomes 1, 2, and 3 years after CI pertaining to the questionnaires were documented and analyzed.

**Results:** The patients were divided into symptomatic (10, 37%) and asymptomatic (17, 63%) groups according to with or without facial and vestibular nerve stimulation. The MCL pertaining to the symptomatic group was significantly lower than asymptomatic group, but Categories of Auditory Performance (CAP) scores 1 year after surgery was better (*p* < 0.05). The subjects were divided into flat (12, 44.4%) and curved (15, 55.6%) groups based on the contour of MCL map. The MCL and D were lower and shorter in the curved group than the flat group, and CAP score 1 year after surgery and Speech Intelligibility Rating (SIR) 3 years after surgery were better (*p* < 0.05).

**Conclusion:** Although abnormal reactions such as facial and vestibular nerve stimulation were observed to be more frequent, lower MCL and better outcomes were observed in relation to the shorter D.

## Introduction

Common cavity deformity (CCD) is a congenital inner ear malformation characterized by the fusion of cochlea and vestibule forming a cavity, which is usually attributed to the arrested otocyst maturation during the fourth week of embryonic development (1). On account of the characteristic anatomy associated with CCD, the distribution of spiral ganglion, which is located on the outer wall of the cavity, differs from that of normal cochlea (2). Cochlear implantation (CI) is an effective mode of treatment of CCD, which stimulates the spiral ganglion to facilitate the reception of stimuli (3) and effects improved outcomes. The recommended surgical approach for CI to position the electrodes in close proximity to the cavity wall is the transmastoid slotted labyrinthotomy approach (TSLA) with customized electrodes (4). However, previous studies have reported that some CCD patients underwent CI experienced facial nerve stimulation, or nystagmus during the implant activation and programming procedures (5), owing to the stimulation of facial or vestibular nerves. Consequently, the audiologist had to alter the stimulation level or deactivate the electrodes responsible for the reactions, which implies that the current scenario warrants further investigation regarding the appropriate position of cochlear electrode. To the best of our knowledge, postoperative programming after CI is vital to the process of rehabilitation, especially in patients with inner ear malformations (6). The factors that are essential to attain ideal performance include appropriate maximum comfortable level (MCL), threshold level (TL), evoked compound action potential (ECAP), frequency allocation, etcetera (7).

Postoperative radiographic evaluation is an effective method that can be employed to perceive the cochlear electrode and radiographic techniques such as the transorbital X-ray, digital volume tomography, and computed tomography (CT) scans have been employed for the same (8). Three-dimensional (3D) reconstruction techniques such as volume rendering (VR) have been used to demonstrate the complex inner ear anatomy and postoperative evaluation of the cochlear implant configuration, owing to the stereo structure of the cochlea (9). The multiplanar volume reconstruction (MPVR) technique is a reliable and direct technique that can be employed for multidirectional perception of hollow viscera (10) and measurement of the same. Hence, it may be a suitable option for the evaluation of CCD. In addition, literature does not include any similar reports on the subject and no previous study has investigated the application of the aforementioned techniques in the same context.

Hence, the present study attempted to analyze the radiographic measurements, cochlear programming parameters, and postoperative performance of CCD patients, in order to determine the most appropriate strategy for electrode implantation.

## Methods

### Participants

The present study included a total of 25 patients (27 ears) with CCD who underwent CI during the time period from January 2016 to September 2020 at our institution. This study was approved by the Institutional Review Board of our center. All the patients underwent preoperative high-resolution computed tomography (HRCT) and inner ear magnetic resonance imaging (MRI) scans and the diagnosis of CCD was confirmed by two or more physicians from the Departments of Radiology and Otorhinolaryngology. The inclusion and exclusion criteria are stated as follows: (1) subjects with bilateral severe or profound sensorineural hearing loss, (2) willingness to cooperate with the postoperative follow-up, and (3) no severe systemic diseases or intellectual disabilities.

### Routine Clinical Investigations and Surgical Approach

**Figure 1:**
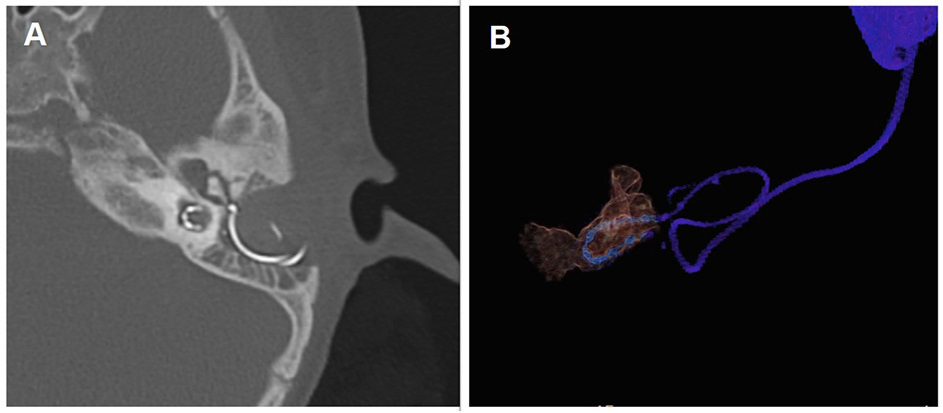
Post-operative computed tomography (CT) scan showing electrodes in CCD patients. **(A)** CT scan of the temporal bone with electrode in the axial position; **(B)** Three-dimensional reconstruction of the electrode and common cavity.

All the participants underwent a routine otorhinolaryngological examination, followed by audiological evaluation and preoperative CT and MRI scans. The patients underwent CI with customized electrodes (MED-EL Medical Electronics, Innsbruck, Austria) by means of the transmastoid slotted labyrinthotomy approach (TSLA), in accordance with the technique described in previous literature (4). A curved labyrinthotomy was performed and the electrode was positioned within the cavity and filled with muscle. The electrode placed within the common cavity is shown in Figure 1. None of the patients displayed any postoperative complications such as facial paralysis or cerebrospinal fluid leakage.

### Post-operative Radiographic Image Acquisition and Post-processing

**Figure 2:**
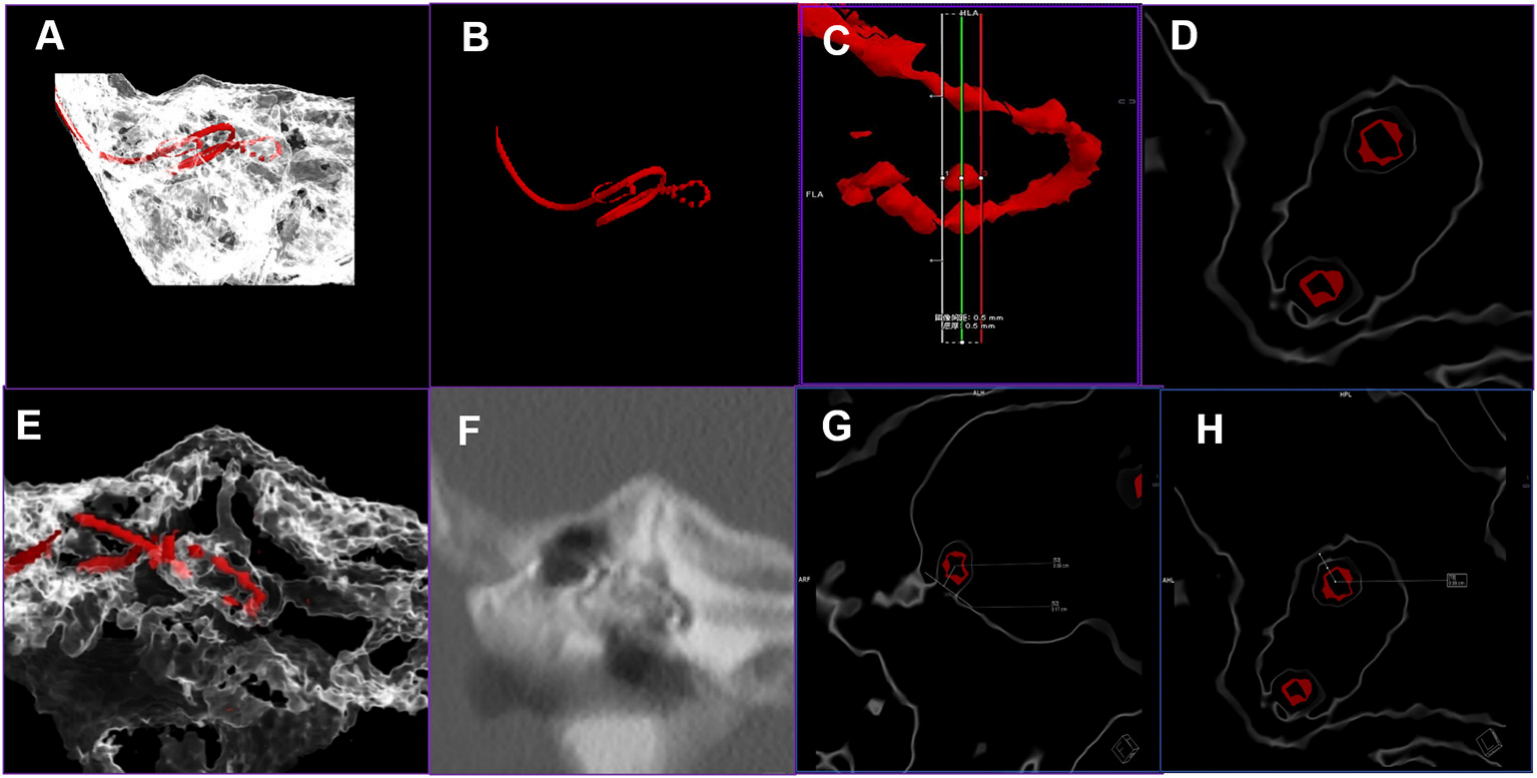
The procedure of volume rendering (VR) and multiplanar volume reconstruction (MPVR) technique for the evaluation of the distance between electrodes and cavity wall. **(A)** The VR image of electrode and temporal bone; **(B)** 3D image of the electrode after the other structures are concealed; **(C)** The layers are located and selected on the 3D image; **(D)** MPVR images were reconstructed and the second layer was selected to measure the distance; **(E)** The thick layer of MPVR images; **(F)** The multiplanar reconstruction images; **(G)** The method of evaluation of the distance between the electrode and cavity wall in situations involving the fusion of the silica gel sleeve outside the electrode and wall of the common cavity; **(H)** The method of evaluation of the distance between the electrode and cavity wall in scenarios that do not involve fusion of the silica gel sleeve and cavity wall.

All the patients underwent CT scan of the temporal bone 4 days after the surgical procedure using the GE 64-row helical CT scanner (USA). The scanning parameters are stated as follows: a bone algorithm was used; window width: 4,000 HU; window level: 700 HU; voltage: 140 kV; current: 350 mAs; section interval: 0.65 mm; section thickness: 0.65 mm; scanning matrix: 512 × 512; and scanning field: 20-24 cm. The data obtained through CT scans were transferred to a Siemens Force CT syngo *via* VB20 post-processing workstation, in order to perform the post-processing procedures for MPVR and multiplanar reconstruction (MPR). The MPVR images were used to gauge the minimum distance between each electrode and lateral wall of the common cavity. The CT images were opened by means of a combination of the dual-energy and vascular opening methods. Initially, the VR technique was used for reconstruction of images. The current study employed the inner ear red Volume Rendering Technique mode equipped by the post-processing machine for the reconstruction of VR images using double threshold technology with the thresholds of 763-1089 HU and 2670-2995 HU, respectively. The 3D images were reconstructed using a cutting frame (Figure 2A). Consequently, the 3D structures with the threshold of 763-1089 HU were concealed and only the metal electrode was displayed (Figure 2B). Successively, the layers on the 3D image were located and selected for evaluation (Figure 2C). The metal electrode was rotated to the center of the image so that the long axis of a single electrode was oriented horizontally. Subsequently, three layers of MPVR images were reconstructed perpendicular to the long axis of the single electrode (the positioning lines of the first and third layers were located on either sides of the electrode). The current study selected the second layer to perform the evaluation. The thickness and interval of the reconstructed layer were 0.5 mm and the reconstructed field of view (FOV) was 10 × 10 mm (Figure 2D). The layer thickness and interval were adjusted to 5 and 0.5 mm, respectively, in order to observe the 3D structure of the cochlea, and the FOV was 40 × 40 mm (Figure 2E). The display mode was changed to MPR for comparation (Figure 2F). Finally, the distance between the center of a single electrode and the lateral wall of the common cavity on the same layer was determined. The present study encountered two noteworthy scenarios during the process of measurement that are stated as follows: (1) In situations wherein the silica gel sleeve outside the electrode and the side wall of the common cavity are fused, a line should be made at the edge of the fusion site and the vertical distance from the center of the electrode to the line should be measured, as shown in Figure 2G; (2) If the silica gel sleeve is not fused with the side wall, the minimum distance between the center of the electrode and cavity wall should be assessed directly, as shown in Figure 2H. The same radiologist evaluated the distance between each electrode and cavity wall three times and the average of the three measurements was recorded as the distance of the electrode.

### Post-operative Equipment Programming and Auditory/Speech Evaluation

The devices pertaining to all the patients were activated 1 month after the surgical procedure. Subsequently, the subjects were under regular follow-up with frequent check-ups at 2, 3, 6, 12, 18, 24, and 36 months after the surgery. The patients were permitted to adjust the equipment programming schedule in accordance with their individual conditions. The programming methods are stated as follows: the extracorporeal speech processor of CI was connected to the computer, MAESTRO 8.0.4 was used to gauge the impedance of all the electrodes, and the MCL and TL of each electrode was recorded. Because MCL is related with TL, we next analyses MCL only. Moreover, the current study observed the patient's behavior during the process of programming and recorded uncomfortable reactions, such as facial nerve stimulation or slightly tilting forth and back.

Furthermore, the patients underwent auditory and speech assessments during each follow-up visit by means of the following scales: Categories of Auditory Performance (CAP; index = 0-7), Speech Intelligibility Rating (SIR; index = 1-5), Meaningful Auditor Integration Scale (for patients in the age group of 3-6 years)/Infant-Toddler Meaningful Auditor Integration Scale (for subjects < 3 years of age) (MAIS/ITMAIS; scale = 0-40), and Meaningful Use of Speech Scale (MUSS; scale = 0-40). Parents or guardians of the infants were asked to answer the questionnaires. The questionnaires were used to evaluate the performance at the activation of CI and 1, 3, 6, 9, 12, 18, 24, and 36 months after the activation. The present study compared the scores obtained 1, 2, and 3 years after the surgical procedure. The patients were divided into symptomatic and asymptomatic groups according to with or without abnormal reactions, and flat and curved groups according to MCL map contour.

### Statistical Analysis

The statistical analyses were performed using SPSS (version 23.0; IBM, Armonk, NY). The present study used student's *t*-tests to compare the following parameters pertaining to the symptomatic and asymptomatic groups as well as the flat and curved MCL map groups: electrode impedance, stimulation levels, distances between the electrodes and cavity wall, and the scores of postoperative performances that was assessed using questionnaires (CAP, SIR, MAIS/IT-MAIS, MUSS). Furthermore, the current study employed the Fisher exact chi-square test to compare the proportion distribution of symptomatic and asymptomatic patients in the flat and curved MCL groups. In the current study, *p* < 0.05 was considered to be statistically significant.

## Results

### General Information

The present study involved 14 (56%) male patients and 11 (44%) female patients. Among the study subjects, nine (36%) and fourteen (56%) patients underwent CI on the right and left sides, respectively, and two patients (8%) underwent bilateral CI. The median age of implantation was 2 years (range, 1–7 years). The speech coding strategies were FS4 (20 ears, 74.1%), HDCIS (4 ears, 14.8%), FS4-p (2 ears, 7.4%), and FSP (1 ear, 3.7%). The findings of the postoperative 3D reconstruction CT scan are stated as follows: no electrodes were located in the internal auditory canal (IAC), seven (28%) ears attained complete implantation, and one or more electrodes located outside the common cavity were detected in the other 20 cases. The extra electrode was mainly located at the head or tail end of the customized array.

### Abnormal Reactions During Programming

The present study observed abnormal reactions during cochlear programming in 10 ears (37%), whereas the other 17 ears (63%) did not exhibit any abnormal reaction. Among the aforementioned cases of abnormal reactions, facial nerve stimulation was observed in six cases and four displayed slightly tilting forth and back, owing to vestibular nerve stimulation. The facial nerve stimulation was instigated by the no. 5 electrode in one case, no. 7 electrode in another, and nos. 6, 7, 1, 11, and 12 electrodes in one case. Furthermore, deactivation of the responsible electrodes resulted in resolution of the symptoms. Abnormal reactions presented as angulus oris spasm in the other three cases with facial nerve stimulation and modification of the electric current resulted in resolution of the symptoms. The abnormal reaction of slightly tilting forth and back was caused by the respective no.4, no.5, and no.7 electrodes in three cases. The modification of electric current resulted in resolution of symptoms.

**Figure 3:**
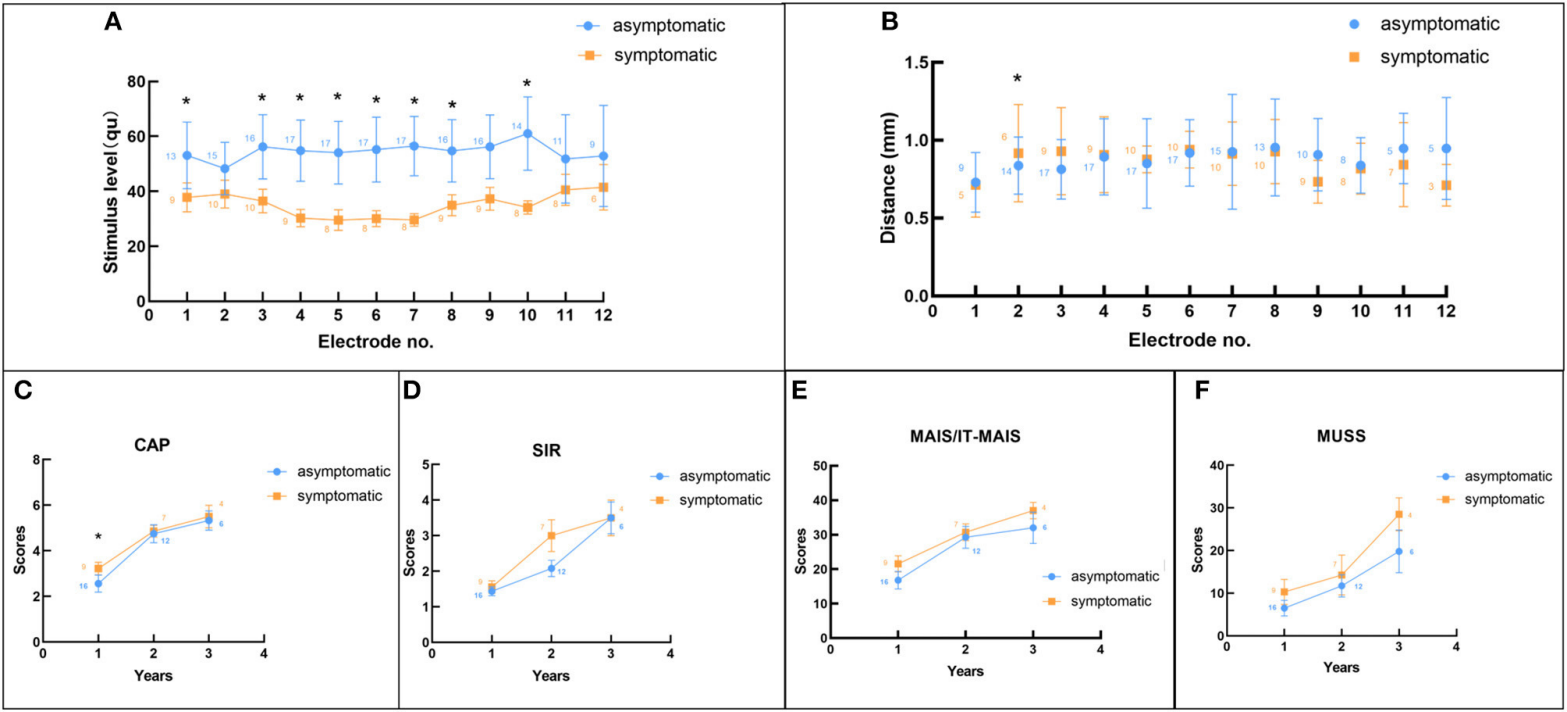
The comparison between symptomatic and asymptomatic groups. **(A)** Comparison of the maximum comfortable level between the two groups; **(B)** Comparison of the distance between electrode and cavity wall pertaining to the two groups; **(C–F)** Comparison of the CAP, SIR, MAIS/IT-MAIS, and MUSS scores 1, 2, and 3 years after surgery in the two groups. The asterisk (*) indicates significant difference between the two groups and the numbers in each legend indicate the sample number.

Moreover, the current study evaluated the impedance and MCL 6 months after the surgical procedure, and the distance between each electrode and common cavity wall and auditory and speech performance 1, 2, and 3 years after the surgical procedure. The present study performed a comparison of the parameters pertaining to the symptomatic and asymptomatic groups using the independent samples *t*-test. The average distance between the electrodes and common cavity wall in the symptomatic group was observed to be shorter. However, the difference was not statistically significant. In addition, the average MCL in the symptomatic group was significantly smaller (*p* < 0.05) and Table 1 showed the specific datum. The current study did not observe any significant difference between the two groups with regard to the impedance of each electrode. There was a significant difference between the two groups in regard to the MCL of electrodes (no. 1 and nos. 3-10), which was observed to be lower in the symptomatic group compared to the asymptomatic group (Figure 3A). Regarding the distance between each electrodes and common cavity wall, the distances concerning no. 1, 7-12 electrodes were shorter in the symptomatic. However, the difference was not statistically significant (*p* > 0.05), with the exception of the distance concerning no. 2 electrode that was significantly shorter in the asymptomatic group (Figure 3B). Regarding the postoperative auditory and speech results, the CAP scores obtained 1 year after the surgical procedure was observed to be significantly better in the symptomatic group, whereas no significant difference was observed in relation to the scores of other questionnaires (Figures 3C–F).

**Table 1:** The sample size, average impedance, distance between the electrode and cavity wall, and maximum comfortable level (MCL) in the symptomatic and asymptomatic groups.

**Groups**	**Asymptomatic**	**Symptomatic**	***T*-test value**
No.(%)	17(63%)	10(37%)	-
Average impedances	7.65 ± 2.04	7.78 ± 2.17	0.03, 0.957
Average distance	0.89 ± 0.13	0.87 ± 0.1	0.009, 0.926
Average MCL	57.06 ± 11.36	36.19 ± 4.03	7.044, 0.014*

The asterisk (*) indicates significant difference.

### MCL Map Contour

**Figure 4:**
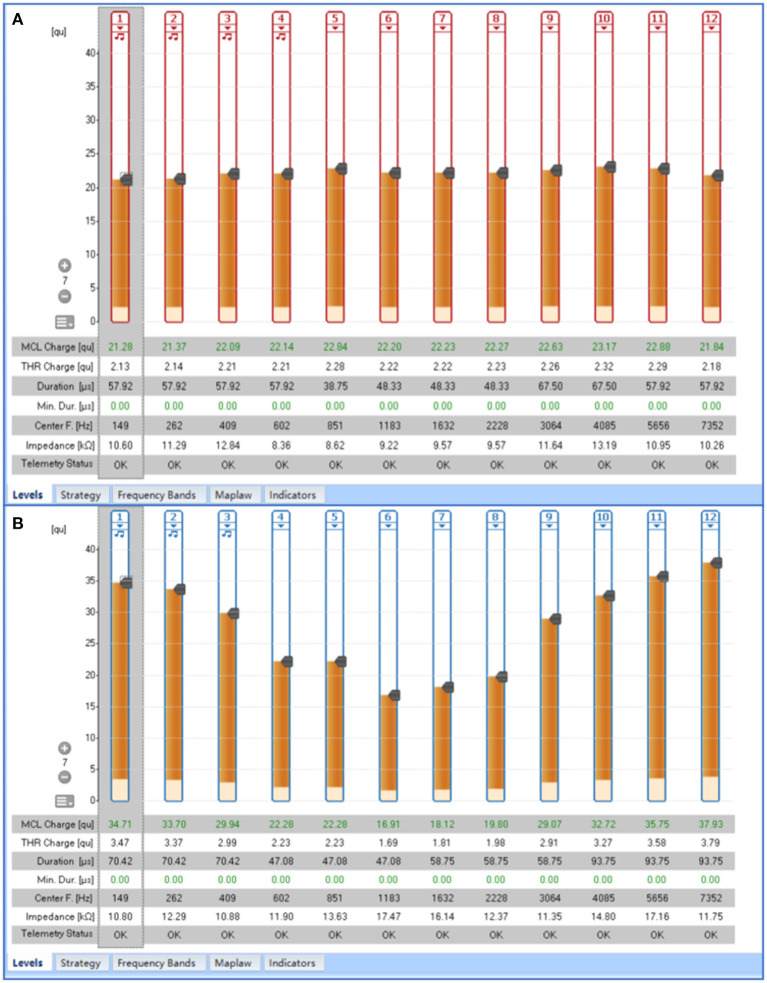
The contour of the maximum comfortable level (MCL) map. **(A)** Flat contour of the MCL map; **(B)** Middle-curved contour of the MCL map.

**Figure 5:**
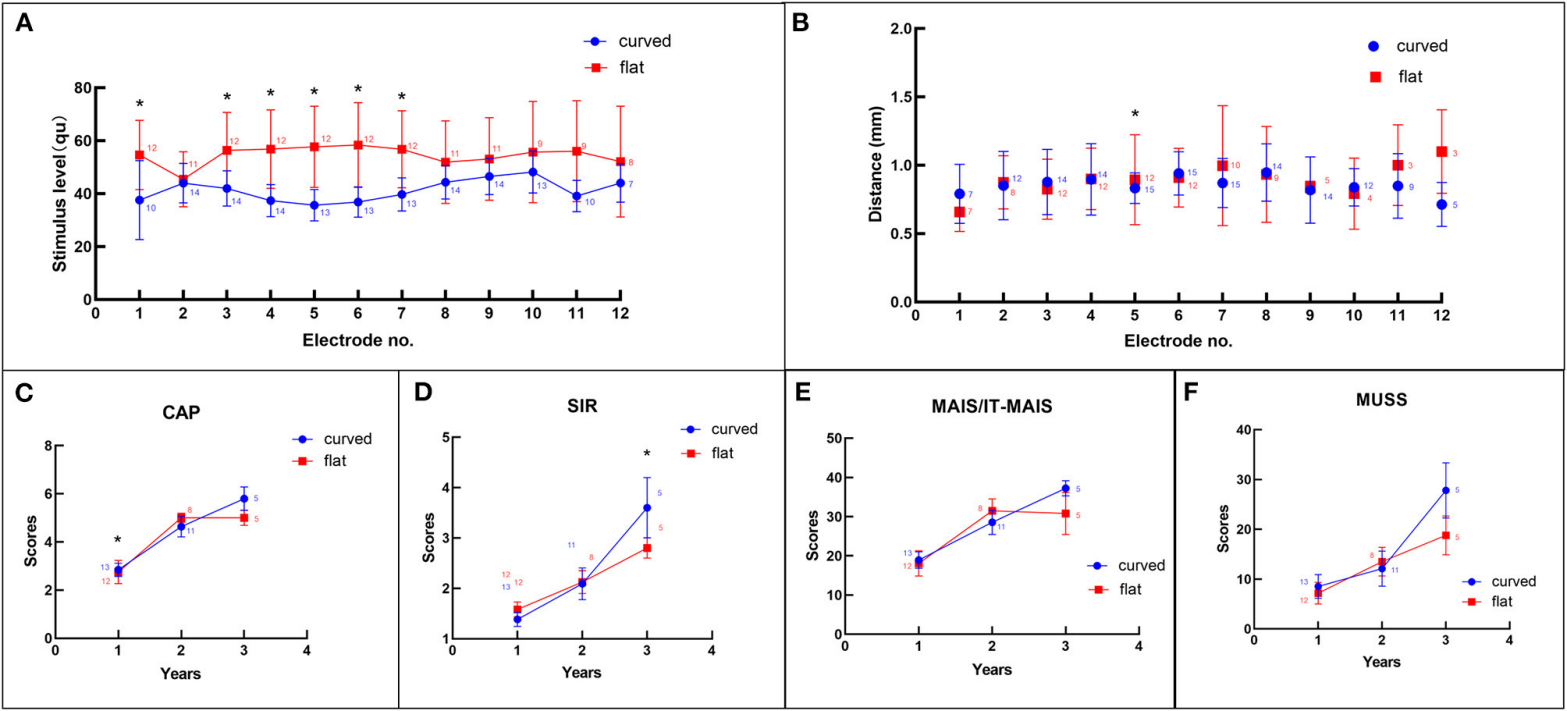
Comparison between flat and curved groups. **(A)** Comparison of the maximum comfortable level between the two groups; **(B)** Comparison of the distance between electrode and cavity wall in the two groups; **(C–F)** Comparison of the CAP, SIR, MAIS/IT-MAIS, and MUSS scores 1, 2, and 3 years after surgery in the two groups. The asterisk (“^*^”) indicates significant difference between the two groups and the numbers in each legend indicate the sample number.

In the present study, the contour of the MCL map was flat in relation to 12 ears (44.4%), which implied comparable MCL with reference to each electrode (Figure 4A), and curved in relation to 15 ears (55.6%), which indicated lower levels of stimuli in relation to the electrodes in the middle of the array and higher levels of current in relation to the electrodes located at the top and end sides of the array (Figure 4B). In the flat group, merely one ear showed an abnormal reaction during programming, whereas nine ears (69%) in the curved group were symptomatic. Fisher exact chi-square test revealed that there was significant difference between the two groups (χ = 7.349, *p* = 0.007). The average distance between the electrode and cavity wall was shorter in the curved group, compared to the flat group. Moreover, the average MCL was observed to be lower in the curved group than the flat group. However, the difference was not statistically significant and the MCL was significantly different (Table 2). The present study did not observe any significant difference between the two groups with regard to the impedance of each electrode. The MCL in the flat group was observed to be higher, compared to the curved group (Figure 5A). Moreover, the present study observed significant difference between the two groups in regard to the MCL in relation to the nos. 1, 3, 4, 5, 6, and 7 electrodes (*p* < 0.05) and the flat group displayed higher. Regarding the distance between each electrode and cavity wall, the distances of nos. 2, 4, 5, 7, 9, 11, and 12 electrodes were observed to be longer in the flat group, compared to the curved group. Additionally, the two groups displayed significant difference with reference to the distance of the no. 5 electrode (Figure 5B). The current study observed significant difference between the two groups in regard to the CAP after 1 year score and SIR score 3 years after surgery. The curved group displayed better outcomes, compared to the flat group (Figures 5C-F).

**Table 2:** The sample size, average impedance, distance between the electrode and cavity wall, and maximum comfortable level (MCL) in the curved and flat groups.

**Groups**	**Curved**	**Flat**	***T*-test value**
No.(%)	15(55.6%)	12(44.4%)	-
Average impedances	7.56 ± 1.99	7.9 ± 2.19	0.306, 0.585
Average distance	0.86 ± 0.08	0.91 ± 0.15	2.620, 0.118
Average MCL	42.34 ± 5.90	58.06 ± 15.17	6.586, 0.017*

The asterisk (*) indicates significant difference.

## Discussion

CCD is a severe inner ear malformation with anatomical variability. A previous study has reported that the volume of common cavity ranged from 52.48 to 233.40 mm^3^ (11). Consequently, implementation of the perfect electrode placement is a challenging endeavor. Moreover, previous studies have reported certain clinical scenarios involving the detection of extracochlear electrodes (3). In the present study, assessment of the 3D radiographic images revealed that only seven sides achieved complete implantation. However, as per the clinical records, complete implantation was documented in relation to 20 sides and the difference was mainly instigated by the electrodes at the top and tail ends of the array, which are close to the entrance of the cavity. This may be attributable to the visual error regarding the entity and 3D images. Furthermore, the radiographic images may not display the wall of the cochleostomy site clearly and it can be distorted or clouded by metal artifacts and the filled muscle can cause confusion. In addition, the speech coding strategy employed in 74.1% of the cases in the current study was FS4, which has a wider range of frequencies information, compared to HDCIS (12) and FSP (13). This further indicates that a wide range of fine structure information can be employed in the patients with CCD and that the 3D images may exaggerate the number of extra-cochlear electrodes.

It is known that the abnormal reaction of nerve stimulation during cochlear programming is more frequent in patients with CCD, compared to the patients with normal cochlea. The common symptoms include facial and vestibular nerve stimulation and corresponding responses (5). In the present study, 37% of the subjects displayed abnormal reactions during programming, among which, facial nerve stimulation was observed in 60% of the cases and 40% exhibited vestibular nerve stimulation and slightly tilting forth and back. The electrodes that instigated the reactions were mainly located in the middle of the electrode arrays, such as nos. 4-7 electrodes. The current results are concurrent with the incidence of facial nerve stimulation reported by previous studies. For instance, the rates of facial nerve stimulation reported by Beltrame et al. (3) and McElveen et al. (14) were 4/16 and 1/4, respectively. In addition, the aforementioned studies observed that the electrodes that triggered the abnormal reaction were located medially (nos. 4, 5, 6, 7, 8 electrodes). Regarding the vestibular symptoms, literature includes only a single case report by Sennaroglu et al. (5) which reported that the patient developed nystagmus during implant activation and the phenomenon was considered to be attributable to the common vestibulocochlear nerve stimulation. In the current our study, four cases exhibited slightly tilting forth and back during the activation of electrodes and the electrodes that instigated the reaction were the medial electrodes, which were mainly located near the IAC. However, in some subjects with facial nerve stimulation, the reaction was instigated by electrodes located at the top and tail ends (nos. 1, 11, and 12). The abovementioned results can be attributed to the anatomy of the nerve course. The sites of labyrinthotomy and electrode insertion near the IAC are close to the facial nerve and the incidence of aberrant facial nerve is high among patients with CCD (14), which is another reason for the occurrence of facial nerve stimulation.

The current study analyzed the differences between symptomatic and asymptomatic groups with regard to the electrode impedance, magnitude of electric current, distance between electrode and cavity wall, and performance outcomes, in order to understand the underlying cause of the abnormal reactions and to facilitate the procedure of CI in clinical settings. To the best of our knowledge, no previous study has investigated the programming and radiology parameters in CCD patients. Additionally, the current sample size is larger, compared to the previous studies regarding the same (15, 16). The present results revealed that the average distance and MCL pertaining to the symptomatic group were lower, compared to the asymptomatic group. The results indicate that the shorter the distance between electrode and cavity wall, the lower the MCL and higher the likelihood of the incidence of abnormal reactions. This may be for the reason that if the electrode is closer to the cavity wall, the magnitude of electric current must be reduced to avoid uncomfortable sensations. As for each electrode, the MCL of nos. 1, 3-10 electrodes were significantly higher in the asymptomatic group, compared to the symptomatic group. The distances were greater in the asymptomatic group, compared to the symptomatic group. However, the difference was not statistically significant, except in relation to the no. 2 electrode (significantly longer in the symptomatic group). The abnormal condition with reference to the no. 2 electrode may be the result of physical factors. When the electrode array was pushed closer to the cavity wall, the medial electrodes were pushed to a greater extent, and the no. 2 electrode was positioned barely into the cavity and a little bit farther from the wall. Individual electrode analysis revealed lower MCL in relation to the medial electrodes, which was consistent with the characteristics of the electrodes that triggered the symptoms, as discussed previously. However, the scores of the questionnaires revealed that the symptomatic group was better than the asymptomatic group in both speech and auditory performances, which implied that the shorter the distance between electrode and cavity wall, the better the outcomes. The aforesaid results might be attributable to the fact that spiral ganglion cells receive more electric information at closer distances, which is consistent with the advantage of the perimodiolar electrode that is located adjacent to the modiolus (17). In addition, the electrical current may decline if the electrodes are placed adjacent to spiral ganglion cells, thereby reducing the power consumption and increasing speech perception (17). In the current study, significant difference between symptomatic and asymptomatic groups was observed only in regard to the CPA score 1 year after the surgical procedure, there was no significant difference in the SIR score between the two groups, and the CAP score was greater than the SIR score, which may be for the reason that the auditory ability (evaluated by CAP) developed at a faster rate than the ability of speech (evaluated by SIR) during the 1 year after the surgical procedure. Previous literature has reported similar conclusions. For instance, Guo et al. and Lye et al. studied 544 children who underwent CI and observed a rapid improvement in CAP scores within 24 months after the surgical procedure and rapid improvement in the SIR score was observed during the time period from 12 to 24 months after the surgery (18). However, the study did not observe any significant difference in regard to the performance after 2 and 3 years after the surgery, which may be attributed to the limited sample size (the duration of follow-up was not adequate in some patients; Figure 3). Furthermore, the results illustrated that despite the incidence of abnormal reactions, proper adjustment can result in good and promising outcomes.

The present study divided the cases into two groups on the basis of the contour of MCL maps of the 12 electrodes, in order to further investigate the effect of stimulation levels on the postoperative performance. The aforementioned categorization was performed in view of the observation of middle-curved contour (the MCL of medial electrodes were lower than that of the marginal electrodes; Figure 4) of the MCL map in certain patients (as observed during the process of cochlear programming). As the programming parameter generally tends to be stable for a duration of 6 months after the surgery (19), the present study selected the MCL maps pertaining to the same time period for analysis. In the present study, the MCL maps corresponding to more than half the subjects displayed curved contours, which further indicated the characteristic nature of the programming procedure associated with CCD. Furthermore, most of the subjects in the curved group were symptomatic, which may be attributed to the fact that the curved shape was caused by the occurrence of abnormal reactions. Generally, in the event of uncomfortable reactions, the MCL may be adjusted to a lower level. In addition, the electrodes responsible for the abnormal reactions are usually located medially, which results in the middle-curved shape of the MCL map. The MCL and average distance between the electrode and cavity wall in the curved group were observed to be lower than those in the flat group, but the distances different weren't significantly, which may be due to the smaller sample size and larger individual variation. An additional reason is the diminutive nature of the distance between the electrodes and cavity wall, usually <1 mm, and slight deviations may lead to considerable differences. For each electrode, the present study observed significant difference between the two groups in relation to the MCL concerning nos. 1, 3, 4, 5, 6, and 7 electrodes and the distance with reference to no. 5 electrode. The postoperative performance, evaluated by means of scores obtained using questionnaires, was observed to be better in the curved group, compared to the flat group. Hence, it can be concluded that the distance were shorter, the magnitudes of electric current was lower, and the outcomes were better. The results can be elucidated on the basis of the regional anatomy, that is, the electrode array was placed arcuately within the cavity in the present research. The medial electrodes (nos. 4-7) were usually located near the IAC, which comprises nerves including the cochlear, facial, and vestibular nerves. Greater proximity to the cochlear nerve effects better outcomes, whereas proximity to the facial and vestibular nerves generates frequent abnormal reactions. A previous study by Yamazaki et al. (20) reported that the electrically evoked auditory brainstem responses (EABR) rate concerning medial electrodes was superior in CCD patients, which confirmed the fact that the medial electrodes provide important auditory information.

Moreover, the present study initially applied the MPVR technique for the evaluation of the temporal bone, which is mainly employed in the analysis of hollow viscera, such as the trachea, esophagus, and blood vessels (10). MPVR is a 3D reconstruction method that can yield images from any angle and remove unwanted structures through the application of varying thresholds. The inner ear has complex structures and overlapping positions and the MPVR offers continuous observation of the electrodes within the cavity from any angle on the 3D images. Additionally, the MPVR images offer sharper edges with less distortion (see Figures 2E,F), compared to conventional MPR images. The thick layers in MPVR images can provide more information in 3D (see Figure 2E) and the thin-layer MPVR images can display the actual structure of the electrodes clearly (see Figure 2D). The reconstructed images obtained using the MPVR technique can be used to evaluate the distance. The customized electrodes include a silica gel sleeve around the metal electrode and clear display of the relationship between electrodes and wall cavity is difficult on conventional MPR images, whereas thin-layer MPVR images can clearly demonstrate if the electrode is adhered to the common cavity wall.

The current study has certain limitations. The direct correlation between the distance of the electrode from the cavity wall and outcomes, and the optimal distance between the electrode and cavity wall were not determined, owing to the limited sample size and diverse shapes of the cavities. Hence, the current study performed a comparison by means of categorization based on clinical symptoms.

## Conclusion

More than one-third of the patients with CCD who underwent TSLA with customized electrodes displayed abnormal reactions, such as facial and vestibular nerve stimulation, and the symptoms were mainly instigated by medial electrodes. The comparison revealed the MCL was lower, the distance between electrodes and cavity wall was shorter, and the outcomes were better in symptomatic patients. In addition, the MCL maps displayed curved contours in more than half of the patients and the MCL and distance pertaining to the curved group were lower than those in the flat group. However, the postoperative outcomes in the curved group were better than the flat group. The results indicated that although abnormal reactions were observed to be more frequent, better outcomes were observed in relation to the shorter distance between the electrodes and cavity wall.

## Data Availability Statement

The original contributions presented in the study are included in the article/supplementary material, further inquiries can be directed to the corresponding author/s.

## Ethics Statement

The studies involving human participants were reviewed and approved by Ethics Committee of Beijing Tongren Hospital. Written informed consent to participate in this study was provided by the participants' legal guardian/next of kin.

## Author Contributions

XW mainly wrote the manuscript. HZ did radiology measurement and wrote the radiology part. SLu and MY did follow-up of the patients. BC and JC collected the clinical and radiology information. LZ helped do statistics. SLi did cochlear programming for patients. JX guided the radiology measurement and discussion. YL did CI surgery and guided the research. YK did cochlear programming and guided the writing of the manuscript. All authors contributed to the article and approved the submitted version.

## Funding

YL received grant from National Natural Science Foundation of China (grant no. 81870716) and Natural Science Foundation of Beijing (grant no. 7212015). XM received grant from Beijing Tongren Hospital (grant no. 2020-YJJ-ZZL-031).

## Conflict of Interest

The authors declare that the research was conducted in the absence of any commercial or financial relationships that could be construed as a potential conflict of interest.

## Publisher's Note

All claims expressed in this article are solely those of the authors and do not necessarily represent those of their affiliated organizations, or those of the publisher, the editors and the reviewers. Any product that may be evaluated in this article, or claim that may be made by its manufacturer, is not guaranteed or endorsed by the publisher.
